# Morphology and Structure of TiO_2_ Nanotube/Carbon Nanostructure Coatings on Titanium Surfaces for Potential Biomedical Application

**DOI:** 10.3390/ma17061290

**Published:** 2024-03-11

**Authors:** Tsanka Dikova, Daniel P. Hashim, Neli Mintcheva

**Affiliations:** 1 Faculty of Dental Medicine, Medical University of Varna, 55 M. Drinov Str., 9000 Varna, Bulgaria; 2 CSS Nanotech Inc., Unit 427, 2368A Rice Blvd., Houston, TX 77005-2652, USA; daniel@cssnanotech.com; 3 Department of Chemistry, University of Mining and Geology, 1700 Sofia, Bulgaria

**Keywords:** titanium and Ti6Al4V alloy, implant coatings, anodization, CVD process, titanium nanotubes, carbon nanostructures

## Abstract

Titanium is the most used material for implant production. To increase its biocompatibility, continuous research on new coatings has been performed by the scientific community. The aim of the present paper is to prepare new coatings on the surfaces of the pure Ti Grade 2 and the Ti6Al4V alloy. Three types of coatings were achieved by applying anodization and chemical vapor deposition (CVD) methods: TiO_2_ nanotubes (TNTs) were formed by anodization, carbon nanotubes (CNTs) were obtained through a metal-catalyst-free CVD process, and a bilayer coating (TiO_2_ nanotubes/carbon nanostructures) was prepared via successive anodization and CVD processes. The morphology and structure of the newly developed coatings were characterized using SEM, EDX, AFM, XRD, and Raman spectroscopy. It was found that after anodization, the morphology of the TiO_2_ layer on pure Ti consisted of a “sponge-like” structure, nanotubes, and nano-rods, while the TNTs layer on the Ti alloy comprised mainly nanotubes. The bilayer coatings on both materials demonstrated different morphologies: the pure Ti metal was covered by a layer of nanotubular and nano-rod TiO_2_ structures, followed by a dense carbon layer decorated with carbon nanoflakes, and on the Ti alloy, first, a TNTs layer was formed, and then carbon nano-rods were deposited using the CVD method.

## 1. Introduction

In medicine, titanium and its alloys are commonly used materials for the production of implants due to their high biocompatibility, bioadhesive characteristics, and mechanical properties similar to those of bone tissue. Titanium materials demonstrate high corrosion resistance resulting from the formation of a robust and protective TiO_2_ layer, which passivates the titanium surface and reduces further metal oxidation [1,2]. The physico-chemical properties of the implant surface, such as roughness, grain size and texture, surface energy and wettability, surface composition, and charge can stimulate osteoblast adhesion and enhance matrix mineralization [3,4,5]. To improve biocompatibility and tune the aforementioned surface properties of biomaterials, various methods have been used: to mention a few, mechanical (grinding and sandblasting), chemical (etching and CVD process), electro-chemical (anodization), physical (laser surface texturing and patterning, PVD process), etc. [2,3,6,7]. Each method demonstrates certain sets of advantages and disadvantages, which is why, in many cases, a combination of two or more techniques is employed to obtain superior properties. For instance, the additional processing gives rise to surface modifications such as higher roughness, porous TiO_2_ layer formation [8,9], complex bi/multilayer coatings [5,7,9], and hierarchical structures on the surface [9,10]. 

The most common approach to improving the biocompatibility of Ti implants is modification of the oxide layer on the Ti surface into a porous nanotubular structure. It is considered that the porous layer mimics the structure of bones, thus enhancing the multiple osteoblast behavior [11,12,13]. The cell response depends on the diameter of the nanotubes (NTs). Small-sized NTs enhance cell adhesion and growth with minimal differentiation, while NTs larger in diameter accelerate the differentiation of mesenchymal stem cells [12]. It has been found that the optimal NT diameter is about 70 nm, which ensures favorable osteoconductivity and osteointegration of the implants [13]. Anodization, which is a rapid, simple, and cost-effective method, is the most preferred approach for producing strongly adherent and highly ordered TiO_2_ NTs on the titanium substrate [11,12,13,14]. During anodization in fluoride-containing solutions, a nanotubular oxide layer was formed on the titanium surface depending on the specific technological regime employed. The NTs dimensions were manipulated by several experimental parameters, including applied voltage, anodization time, electrolyte type, and pH [15]. This allowed for control over the NTs’ diameter, length, and wall thickness [16,17].

In recent years, much attention has been paid towards the application of carbon-based materials such as carbon nanotubes (CNTs) [18,19,20], carbon-based composites [21], diamond-like carbon coatings, and graphene-based materials [22,23] in regenerative medicine. Carbon-based nanomaterials can be embedded into the scaffolds, improving the mechanical properties, or can be coated onto the substrate to alter its surface morphology and to enhance its stability and biocompatibility. The newly-developed carbon nanocomposite coatings, deposited on Co-Cr alloys, have shown higher wear resistance and better biological properties concerning the formation of new tissues in comparison with the uncoated Co-Cr and Ti alloys [21]. The CNTs and graphene-based materials can be easily functionalized to enhance the range of their biomedical applications [18,19,20,22].

The CNTs are attractive biomaterials owing to their unique physical, chemical, mechanical, and biological properties, as well as their high surface area-to-volume ratio [23,24,25,26,27]. CNTs are characterized by excellent electrical and thermal conductivity, robustness, biocompatibility, chemical resistance, high mechanical strength, and lightweight nature [26,27], which make them good candidates for application in all stages of the treatment process for diseases: in diagnosis as biosensing elements, in therapy for targeted drug delivery, in tissue engineering as advanced biomaterials, and additionally in prevention as vaccine delivery vehicles. Interestingly, in tissue engineering, the CNTs are mainly used for the reinforcement of existing scaffold materials or the synthesis of different types of coatings [28]. Haniu H. et al. found that the CNTs possess high biocompatibility and accelerate in vivo osteogenesis [25]. Favorable biocompatibility of vertically aligned super-hydrophobic CNT films were reported by Lobo A. et al. [29]. The surface structure of multi-walled CNTs carpets helps in cell adhesion; hence, they can be considered as potential scaffolds for cell growth and tissue engineering [20]. On one hand, the CNT-based coatings can improve the conditions for the function of the attached cells, and on the other, they can reduce the amount of metal ions released from the implant surface into human body fluids [23]. It has been discovered by many authors that various types of CNTs (functionalized and non-functionalized) can serve as good surface templates for cellular growth and can stimulate the differentiation and proliferation of different types of cells [23,25,26,27].

The aforementioned characteristics of CNTs closely relate to their structure and morphology. The CNTs are one-dimensional nanomaterials which can be represented as rolled graphene sheets built from thousands of sp^2^-hybridized carbon atoms. Depending on the number of graphene sheets, CNTs can be single-walled (SW), double-walled (DW), or multi-walled (MW). The diameter of CNTs varies from 0.4 nm of SW up to 5–100 nm of MW [18,24,30,31]. The most common method for CNT production is chemical vapor deposition (CVD), which is an easily controllable, cost-effective, and time-saving technique. This method is also known as an effective route for the fabrication of carbon-nanotube-coated metal supports. During the CVD process, the substrate, covered with catalyst nanoparticles (Fe, Co, Ni), is placed into a tube furnace, where the gaseous carbon source (methane, acetylene) is passed and heated. Upon the high-temperature decomposition of hydrocarbon, the CNTs nucleate and grow over the catalyst; thus, the metal nanoparticles remain incorporated into the CNTs [24,30,32,33].

If titanium is used as a substrate, the addition of different transition metals reduces its biocompatibility due to traces of metal catalysts in the carbon layer. This is why different purification techniques (chemical, physical or combined) should be used after the preparation step [31]. However, this stage is a time-consuming and can cause changes in the CNTs’ properties. To avoid post-preparative treatment and to keep the titanium implants free of other transition metal nanoparticles, a metal-catalyst-free CVD formation of CNTs on titanium substrate is recommended. However, the literature data regarding CNT synthesis without metal catalysts are relatively scarce. Takagi et al. reported that 5 nm nanodiamond particles act as growth seeds for CNTs in the ethanol CVD process [34]. Porous Al_2_O_3_ and SiO_2_-dispersed Si plates facilitate CNT formation without any other catalysts [35,36,37,38,39]. Huang et al. scratched the SiO_2_/Si wafers, and single-wall CNTs grew on the scratches during CVD at 900 °C [40]. Cai et al. reported the formation of carbon nanotubes on TiO_2_ nanoparticles as catalysts [41]. Successful synthesis of CNTs on the anodized surface of a Ti alloy without a metal catalyst was performed in Dikova’s work, where it was proposed that the TiO_2_ nanopeaks of the rough titanium surface could serve as sites for nucleation and growth of CNTs [31]. This achievement motivated us to investigate the experimental conditions for CNT growth on treated titanium surfaces, and to check the hypothesis that TiO_2_ can assist in CNT growth. 

This overview of the literature has shown that a porous nanotubular oxide layer increases the biocompatibility of titanium implants on one hand. On the other hand, CNTs can stimulate the growth of different kinds of cells. However, there are no data regarding the combination of the two coatings in a bilayer on a titanium surface for potential biomedical applications. 

The aim of the present paper is to prepare new coatings based on TiO_2_ nanotubes (TNTs) and carbon nanostructures (CNSs) deposited on pure Ti Grade 2 and Ti6Al4V alloys, and to characterize their morphologies and structures. Three types of coatings were formed on the surfaces of titanium materials using different methods. The TNTs were obtained through anodization, the CNTs were achieved via the metal-catalyst-free CVD process, and the bilayer coating consisting of TNTs covered by CNSs was prepared using successive processes of anodization and CVD. To the best of our knowledge, the synthesis of carbon nanotubes and carbon nanostructures on titanium surfaces using a metal-catalyst-free CVD process was performed for the first time in the present study.

## 2. Materials and Methods

### 2.1. Sample Preparation

**Figure 1:**
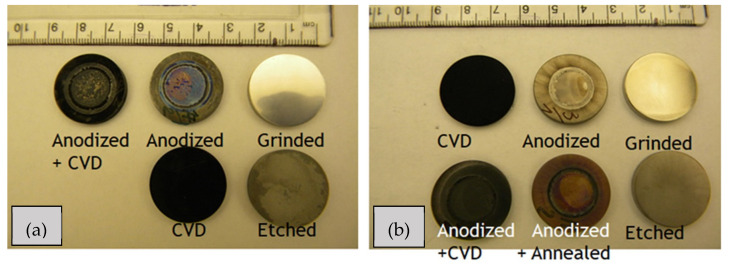
Pictures of samples produced from: (**a**) pure Grade 2 titanium and (**b**) Ti-6Al-4V alloy after different treatments (Note: the anodized and annealed sample of pure Ti is not shown here).

All samples were prepared as round disks with diameters of 24 mm and thicknesses of 3 mm (Figure 1) by using two commercially available materials: pure Grade 2 titanium (99.5%, Sandvik, Stockholm, Sweden) (denoted as Ti metal) and Ti6Al4V alloy with a chemical composition of Al 5.7%, Fe 0.13%, V 3.8%, O 0.089%, and Ti comprising the rest (wt.%) (Sandvik, Stockholm, Sweden) (denoted as Ti alloy). The surfaces of all samples were ground consecutively with 300-, 600-, and 800-grit sandpaper; cleaned in acetone, ethanol, and deionized water via ultra-sonication for 15 min; and then dried with compressed air (Ti metal-ground and Ti alloy-ground). In the next step, these samples were etched in 0.5 wt.% HF acid for 30 min, then immediately rinsed with deionized water and dried (Ti metal-etched and Ti alloy-etched). The etched samples of the two materials were used as controls for comparison of the surface morphology and structure of the newly developed coatings.

Afterward, the samples were separated into three groups depending on the treatment applied.

Group 1 (Anodization)

The Group 1 samples were anodized for 7 h in an electrolyte containing 0.5 wt.% HF acid using a graphite electrode as the cathode and DC power supply. A constant voltage of 25 V was applied for the pure Grade 2 titanium samples and 30 V for the Ti6Al4V alloy. When the anodization was completed, the samples were rinsed with deionized water and dried with compressed air (Ti metal-1′ and Ti alloy-1′). After that, the samples were annealed at 550 °C for 2 h under an argon atmosphere in a tube furnace (Ti metal-1 and Ti alloy-1) (Table 1).

Group 2 (CVD process)

The Group 2 samples (Ti metal-2 and Ti alloy-2) were processed using catalyst-free CVD at 650 °C for 1 h. This was performed in a tube furnace by passing a gas mixture C_2_H_2_:Ar in a volume ratio of 1:5. When the CVD process was complete, the temperature was set to 550 °C and the samples were annealed for 2 h under argon in the same furnace (Table 1).

Group 3 (Anodization and CVD process)

Group 3 (Ti metal-3 and Ti alloy-3) was subjected to a combined treatment. At first, the samples were anodized at the same regime as Group 1, then were treated with the CVD process and annealed like Group 2. The conditions are listed in Table 1.

The technological parameters of anodization (time, voltage, and electrolyte composition) and the CVD process (time, temperature, and carbon career gas) were established after conducting preliminary experiments, and are reported elsewhere [16,31].

### 2.2. Sample Characterization

The surface morphologies of the ground and etched samples of pure Ti and Ti6Al4V alloy, and the samples prepared through treatments (anodization, CVD process, anodization + CVD) were examined via scanning electron microscopy (SEM). The measurements were performed at high voltages of 10 kV, 15 kV, and 30 kV, and the images were taken at different magnifications: 1000, 5 k, 10 k, 20 k, and 50 k. The elemental composition of the studied materials was determined through energy dispersive X-ray spectroscopy (EDX). The micrographs and EDX data were taken using a high-resolution field emission scanning electron microscope, FEI Quanta 400 ESEM FEG (ESEM2) (FEI Company, Hillsboro, OR, USA).

Quantitative measurements of the surface roughness and surface area were obtained by means of atomic force microscopy (Park NX20 Atomic Force Microscope, Park Systems Corporation, Suwon, Republic of Korea). The surface area was measured on a 5 µm × 5 µm horizontal plane. The root mean surface roughness and the average roughness of the samples were calculated. The phase composition was investigated by X-ray diffraction on an XRD analyzer Rigaku D/Max with Cu Kα irradiation (Rigaku, Tokyo, Japan). The Crystallography Open Database was used to determine and assign the peak positions. The carbon nanostructures and TiO_2_ nanotubes were studied by Raman spectroscopy at three different points of the samples’ surface. Graphs were plotted using average values. The spectra were recorded on a Renishaw inVia Raman microscope RL633 Laser (Renishaw plc., New Mills, Wotton-under-Edge, Gloucestershire, UK).

## 3. Results

### 3.1. SEM Investigation

**Figure 2:**
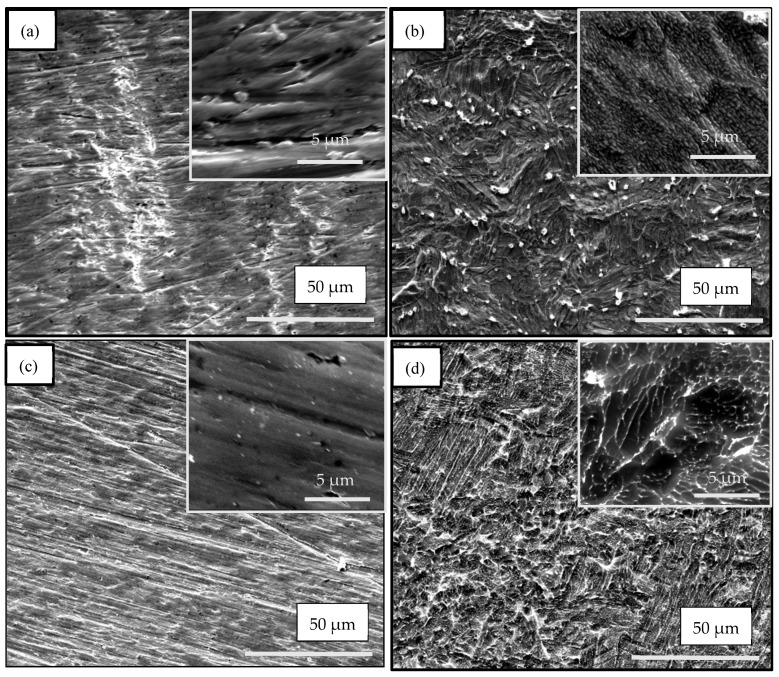
SEM images of the ground and etched samples: (**a**) Ti metal-ground; (**b**) Ti metal-etched; (**c**) Ti alloy-ground; (**d**) Ti alloy-etched (pictures at the right corner show higher magnification (10,000); scale bar: 5 μm).

**Figure 3:**
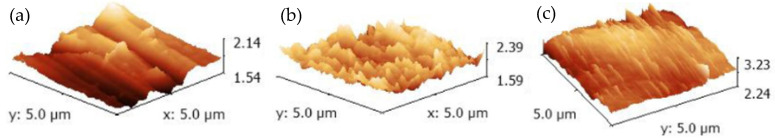
AFM images and quantitative measurement of the surface roughness of: (**a**) Ti metal-ground; (**b**) Ti metal-etched; (**c**) Ti metal-1.

Figure 2 shows the morphology of the ground and etched samples. Traces from the abrasive paper can be seen on the ground surfaces of both materials (Figure 2a,c). After etching, combined micro/nano-roughness and oxide formations were observed on the titanium surface (Figure 2b). The Ti6Al4V alloy surface was characterized mainly by micro/nano-roughness and small amount of oxide situated mostly along the boundaries between the α- and β-phases (Figure 2d). Etching of pure Ti caused slight changes in the micro-roughness, but a significant increase in the nano-roughness (Figure 3). The root mean surface nano-roughness and average roughness of the ground sample (Ti metal-ground) were about 8 nm and 6.5 nm, respectively. These parameters increased by around three times after etching, and reached 23 nm and 17 nm, respectively, for the metal-etched Ti sample. In contrast, after anodization of this material, the nano-roughness decreased, as can be seen in Figure 3c. The root mean surface nano-roughness and the average nano-roughness of Ti-metal-1 were calculated to be 12 nm and 10 nm, respectively. These are approximately two times lower than those parameters for the etched sample (Ti metal-etched).

**Figure 4:**
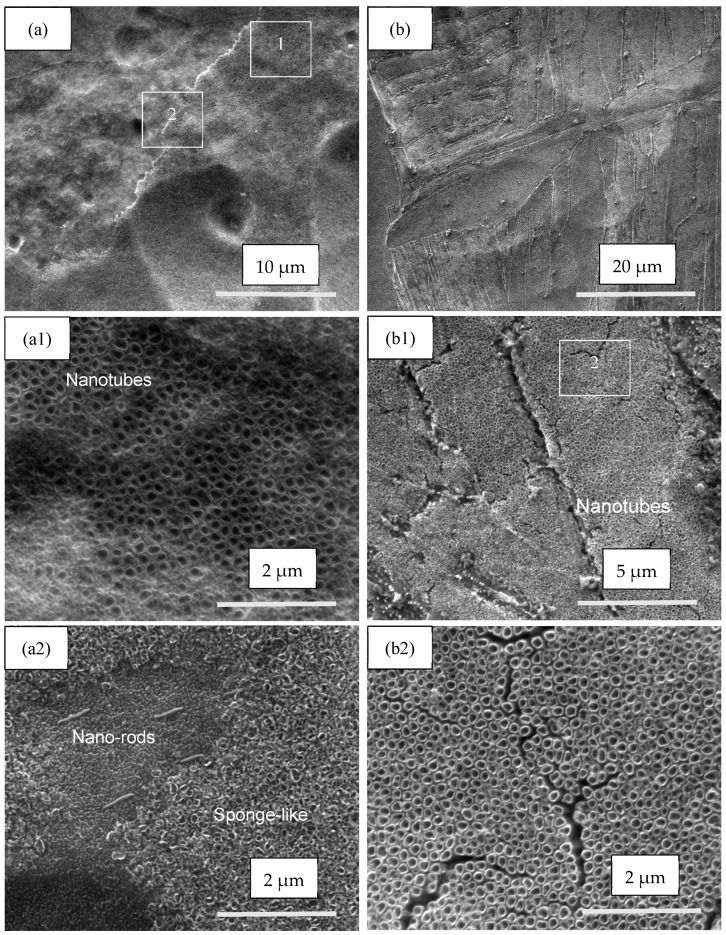
Surface morphology of the materials after anodization and before annealing: (**a**) Ti metal-1′; (**b**) Ti alloy-1′. The images in Figure 4(**a1**,**a2**) were taken at higher magnification of zones 1 and 2. The zone 2 in Figure 4(**b1**) was measured at higher magnification and shown in Figure 4(**b2**).

The morphology of the anodized materials (Ti metal-1′ and Ti alloy-1′) was studied via SEM, and the micrographs are shown in Figure 4. The surface of Ti metal-1′ can be characterized by a large number of pores and craters, with the diameter in the range of 2 µm–25 µm (Figure 4a), indicating an increase in the surface micro-roughness in comparison with ground and etched samples (Figure 2a,b).

Figure 4(a1,a2) (at higher magnification) reveal nanotubular and nano-rod-like structures at a ratio of approximately 70:30, which was found using the SEM tools. The TiO_2_ nanotubular structure consisted of regions with nanotubes and sponge-like areas. The observed TNTs were well-shaped, with an average inner diameter of 120 nm, while the sponge-like area showed pores with irregular shapes and an average inner diameter of 70 nm. The size of the nanostructures was measured based on higher magnification images using SEM tools. The nanotubes/sponge-like ratio was about 50:50.

Anodization of the Ti6Al4V alloy samples led to the formation of well-shaped TNTs with an average internal diameter of 115 nm (Figure 4(b–b2)), which were located mostly on the surface of α-titanium grains. Thus, one can assume that they covered about 90% of the sample’s surface (Figure 4(b1,b2)).

**Figure 5:**
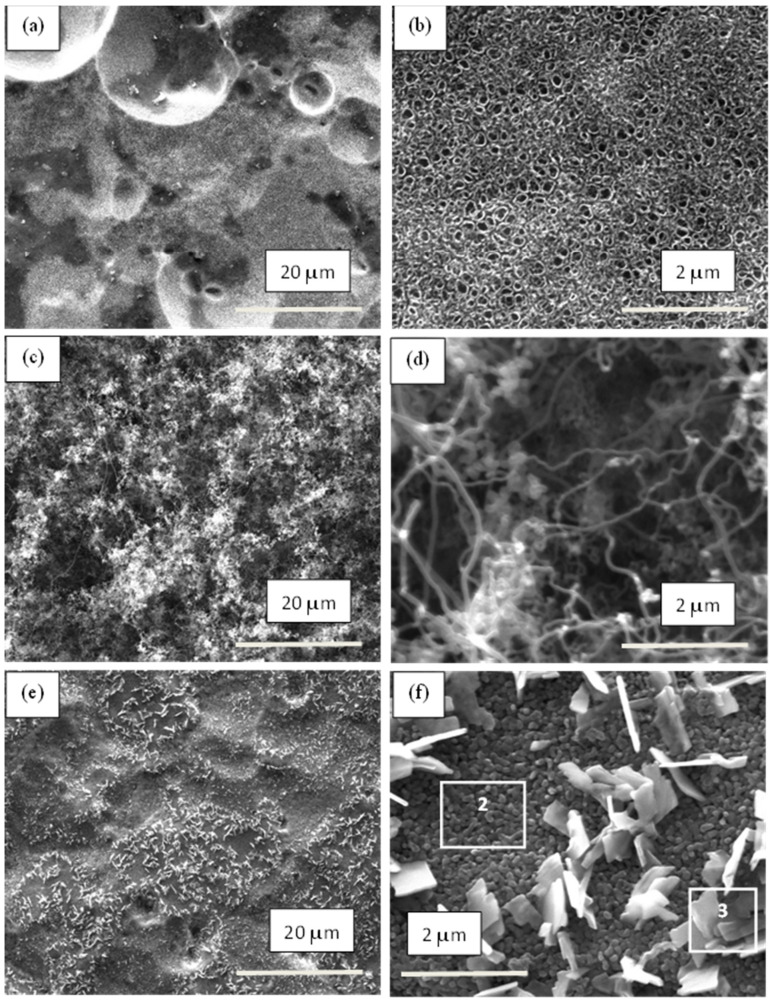
SEM images at different magnifications of the pure Ti samples after three types of treatment: (**a**,**b**): anodization (Ti metal-1); (**c**,**d**): CVD process (Ti metal-2); (**e**,**f**): anodization and CVD (Ti metal-3). The areas 2 and 3 shown in Figure 5f were used for EDX analysis.

**Figure 6:**
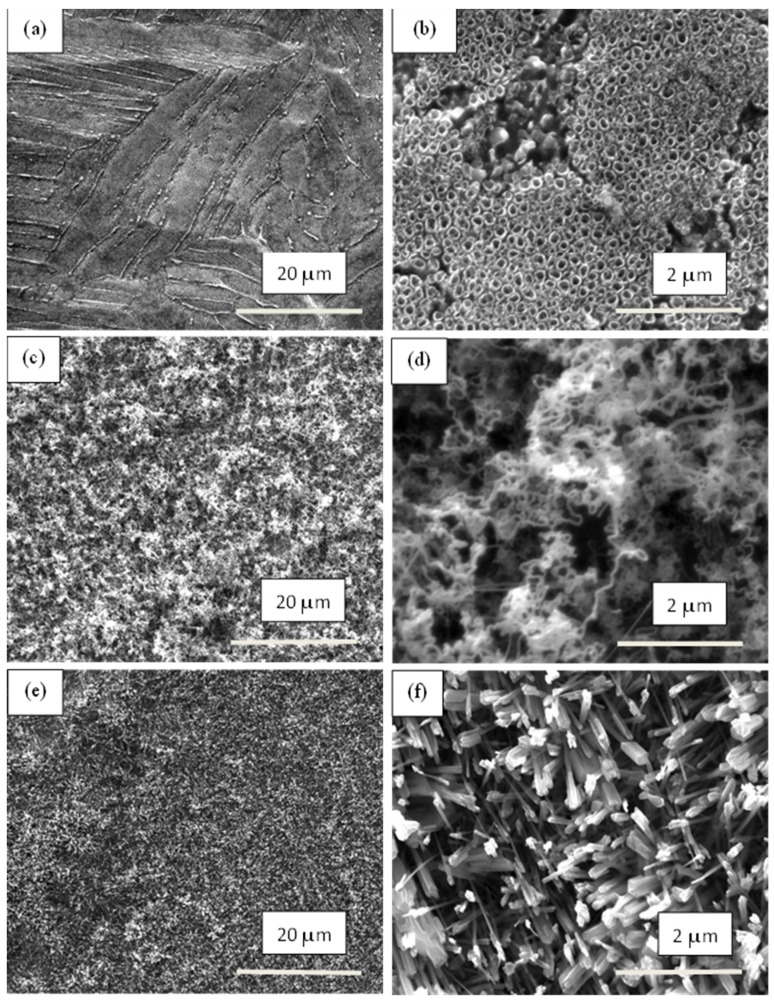
SEM images at different magnifications of the Ti-6Al-4V alloy samples after types of treatment: (**a**,**b**): anodization (Ti alloy-1); (**c**,**d**): CVD process (Ti alloy-2); (**e**,**f**): anodization and CVD (Ti alloy-3).

In the next step of sample processing, the materials were annealed at 550 °C for 2 h, and their morphology was examined again. As can be seen in Figure 5a,b (for Ti metal-1) and Figure 6a,b (for Ti alloy-1), the surface morphology of both samples remained similar to that before annealing, with the exception of the smaller internal diameter (85 nm) of TNTs for Ti metal-1.

Figure 5c,d and Figure 6c,d show the morphology of Group 2 samples (Ti metal-2 and Ti alloy-2, respectively), where long, curly CNTs which formed during the CVD process can be seen. 

The average external diameter of the CNTs was about 75 nm for the Ti metal-2 (Figure 5c,d) and 55 nm for the Ti alloy-2 (Figure 6c,d). The diameter of the CNTs varied in a narrow range between 70 and 85 nm for Ti metal-2, and was even shorter, between 50 and 60 nm, for Ti alloy-2. 

The combination of anodization and the subsequent CVD process gave rise to a bilayer coating on the surfaces of both materials (Ti metal-3 and Ti alloy-3). Their SEM images at different magnifications are presented in Figure 5e,f and Figure 6e,f for Ti metal-3 and Ti alloy-3, respectively. In the case of Ti metal-3, the first layer of the nanotubular and nano-rod structure, formed during anodization, was covered with a dense subsequent layer and carbon nano-flakes (CNFs) grown on its surface (Figure 5e,f). The different orientation of the CNFs hindered the measurement of their sizes. Nevertheless, one can assume their length and height to be about 0.5 µm–1 µm approximately. In contrast, for the Ti alloy-3 sample, the bottom TNT layer was roofed by numerous carbon nano-rods (CNRs) (Figure 6e,f). The estimated diameter of the CNRs varied between 70 nm for single CNRs and 140 nm for aggregated nano-rods. 

### 3.2. EDX Analysis

EDX analysis provided information about the elemental composition of the studied samples. In Table 2 are summarized the EDX data for the atomic percentages of elements on the surface of the Ti metal-1′ in a region with sponge-like morphology, as shown in Figure 4(a2); the Ti alloy-1′ in a TiO_2_ nanotube region, as shown in Figure 4(b2); the Ti metal-3 in different regions (area denoted as 2 and 3), as shown in Figure 5f; and the Ti alloy-3 surface, shown in Figure 6f.

The results reveal that the surface layer of the Ti metal-1′ consisted of titanium and oxygen, showing that the nanotubular structure was made of TiO_2_, while the Ti alloy-1′ showed the presence of the elements Ti, O, Al, and V in accord with the chemical composition of the alloy, suggesting the formation of mixed oxides. Concerning the Ti metal-3 and Ti alloy-3 samples, in addition to the previously mentioned elements, carbon was found as evidence for its formation during the CVD process. The carbon content varied between 14–18% in the different areas (dense layer and nano-flakes) of the Ti metal-3 surface, and was about 20% C on the Ti alloy-3. Therefore, we can conclude that the dense layer and nano-flakes in the Ti metal-3 (Figure 5f) and the nano-rods in Ti alloy-3 (Figure 6f) consisted mainly of carbon; thus, we assume a formation of a carbon layer and CNFs in Ti metal-3 and CNRs in Ti alloy-3 over the initial layer of TNTs.

### 3.3. XRD Analysis

**Figure 7:**
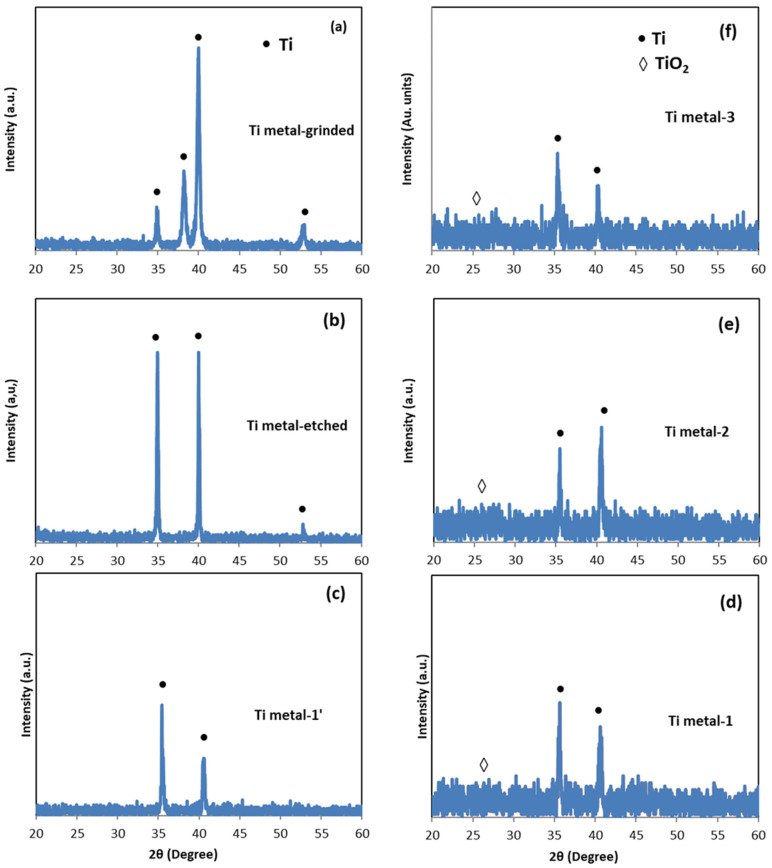
XRD patterns of pure Ti Grade 2 samples after different treatments: (**a**) grinding (Ti metal-grinded); (**b**) etching (Ti metal-etched); (**c**) anodization (Ti metal-1′); (**d**) anodization and annealing (Ti metal-1); (**e**) CVD process (Ti metal-2); and (**f**) anodization and CVD process (Ti metal-3).

**Figure 8:**
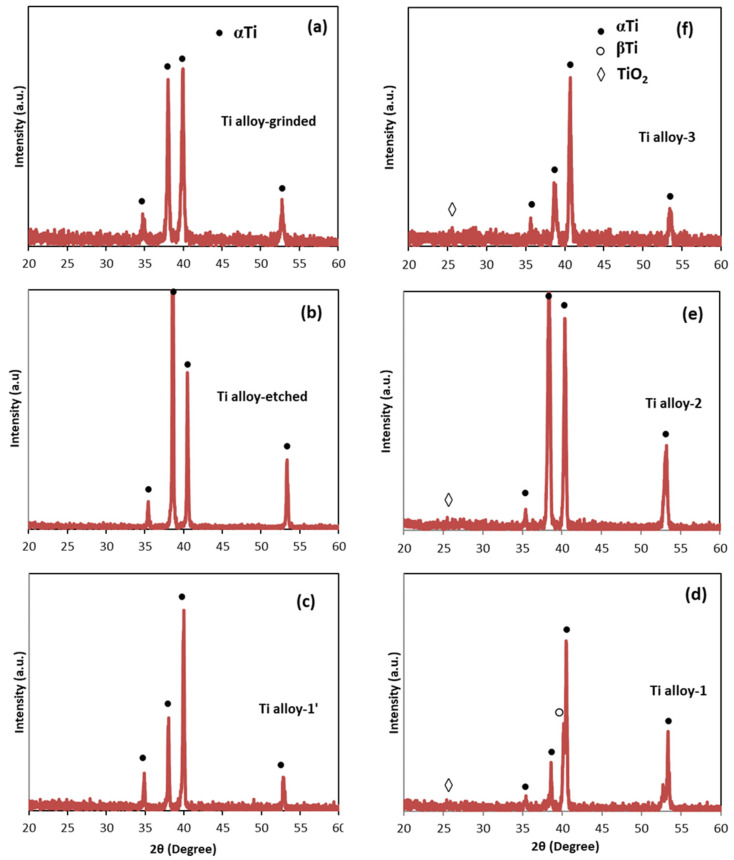
XRD patterns of Ti6Al4V alloy samples after different treatments: (**a**) grinding (Ti alloy-grinded); (**b**) etching (Ti alloy-etched); (**c**) anodization (Ti alloy-1′); (**d**) anodization and annealing (Ti alloy-1); (**e**) CVD process (Ti alloy-2); and (**f**) anodization and CVD process (Ti alloy-3).

The X-ray diffractograms of pure Ti after different treatments are collected in Figure 7. Only peaks assigned for pure Ti metal were registered after the grinding, etching, and anodization steps (Figure 7a–c) [42]. The (002) peak at 2θ = 38.36° disappeared after etching (Figure 7b), and the (102) peak at 2θ = 53.10° was not observed after anodization (Figure 7c–f) due to the formation and growth of the thin oxide layer. In parallel, the X-ray diffractograms of the Ti6Al4V alloy (Figure 8) showed peaks only for the α-Ti phase after treatment by grinding, etching, and anodization (Figure 8a–c) [43]. A weak peak assigned to β-Ti was observed only after annealing of the anodized sample (Ti alloy-1) (Figure 8d).

Weak peaks of TiO_2_ appeared after annealing of the three groups of samples (Group 1, Group 2, and Group 3) of both metal and alloy substrates (Figure 7d–f and Figure 8d–f), indicating a transformation of the amorphous TiO_2_ to crystalline structures [44,45]. Indeed, the intensity of the TiO_2_ peaks was very weak, maybe because of the irregular thickness of the oxide layer and low ratio of TiO_2_ to Ti in the materials. To obtain more information, the presence of TiO_2_ was studied via Raman spectroscopy.

### 3.4. Raman Spectroscopy

**Figure 9:**
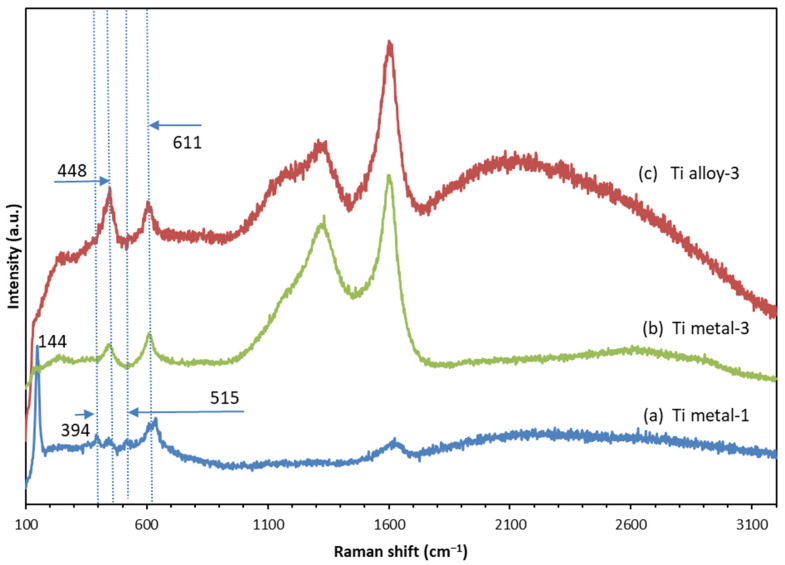
Raman spectra of the samples: (**a**) Ti metal-1; (**b**) Ti metal-3; (**c**) Ti-alloy-3, indicating the peaks of TiO_2_.

All Raman data are listed in Table 3. The Raman spectra of the samples Ti metal-1, Ti metal-3, and Ti-alloy-3 are shown in Figure 9 to highlight the signals of TiO_2_. For the anodized pure Ti (Figure 9a), typical peaks for anatase (144, 394, 515, and 635 cm^−1^) and rutile (442 and 611 cm^−1^) were observed [46]; both were formed after the annealing of the sample. In Figure 9b,c, only two signals at 448 and 611 cm^−1^, assigned to the rutile phase in the samples Ti metal-3 and Ti alloy-3, can be seen. Therefore, at a temperature of 650 °C during the CVD process, a transformation of anatase to rutile, which is a thermodynamically stable form of TiO_2_, took place. These results correspond well with those reported by others [44,45].

**Figure 10:**
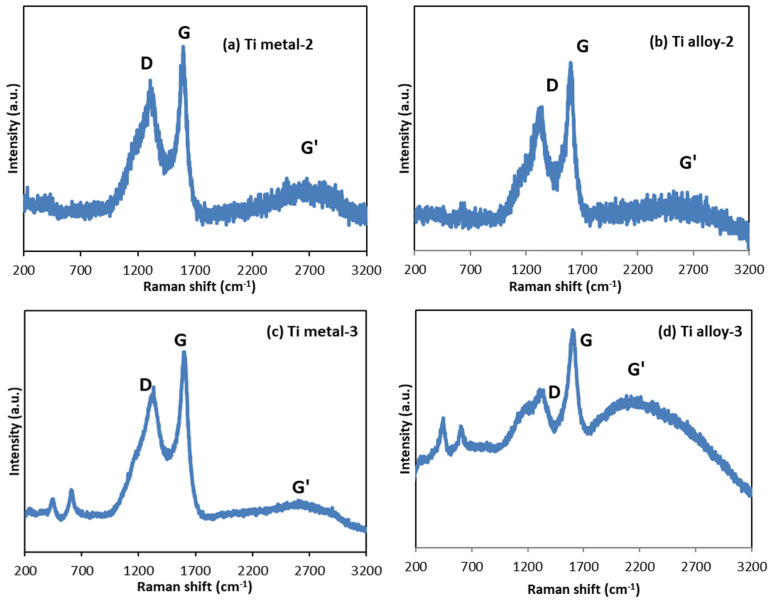
Raman spectra of the samples: (**a**) Ti metal-2; (**b**) Ti alloy-2; (**c**) Ti metal-3; (**d**) Ti alloy-3.

The Raman data of carbon-based materials (Figure 10) are characterized by three peaks in the range 1000 and 3000 cm^−1^, namely a D band at about 1340 cm^−1^, a G band at about 1600 cm^−1^, and a G′ band (also known as 2D) at about 2700 cm^−1^ [20,31,47,48,49]. The D band is associated with the presence of structural defects within the sp^2^-hybridized carbon network, and its intensity relates to the number of defects. The G band comes from the in-plane stretching vibration of the C-C bond in the hexagons of the graphene layer, and is typical for all sp^2^-carbon containing materials such as carbon nanotubes, nanosheets, graphite, graphene, etc. The intensity ratio of the D to G band (I_D_/I_G_) was used for evaluation of the CNTs’ quality. It provided a quantitative estimation of the defect’s density within the graphene plane and the amount of amorphous carbon on the MWCNT walls. The low D/G ratio indicates the low defects, which defines a higher CNT quality [31,48,50].

The Raman spectra showed clear evidence for the formation of CNTs in the Ti metal-2 and Ti alloy-2 samples (Figure 10a,b). The peaks at 1308 cm^−1^ and 1596 cm^−1^ for the Ti metal-2 and 1344 cm^−1^ and 1601 cm^−1^ for the Ti alloy-2 (Table 3) can be assigned to the D and G peaks of CNTs [20,31]. The shapes of the D and G peaks demonstrated the presence of well-structured CNTs [51]. The intensity ratio of the D to G band (I_D_/I_G_) was 0.84 and 0.76 for the Ti metal-2 and Ti alloy-2, respectively (Table 3). Therefore, the CNTs on the titanium alloy surface were characterized by fewer defects than that on the pure Ti metal. The G′ band (or 2D band [50]) was less sensitive to sample defects, but, together with the G-band, the graphitic structure of the CNTs was confirmed [20,51]. In graphene, this band appears as a strong single peak, while in other carbon structures, the shape is modified and broadened [50]. In our case, it appeared as a weak and very wide band centered at 2692 cm^−1^ for the Ti metal-2 and 2650 cm^−1^ for the Ti alloy-2, indicating multilayer structures.

Furthermore, the Raman spectra confirmed the presence of carbon nanostructures on the surfaces of both materials of Group 3 after treatment by anodization and the CVD process (Figure 10c,d). Both the D and G peaks for Ti metal-3 and Ti alloy-3 were more intensive than those of Ti metal-2 and Ti alloy-2 (Figure 10a,b), where the CNTs grew over the etched surface of the pure Ti metal and alloy, respectively. The D, G, and G’ peaks of carbon nanostructures, raised over the titania layer (Ti metal-3 and Ti alloy-3), were detected at 1332 cm^−1^, 1602 cm^−1^, and 2604 cm^−1^ for Ti metal-3, and at 1340 cm^−1^, 1605 cm^−1^, and 2207 cm^−1^ for Ti alloy-3 (Table 3, Figure 10c,d). The I_D_/I_G_ values of these two samples (0.82 for Ti metal-3 and 0.78 for Ti alloy-3) were similar to those of the Group 2 samples (0.84 for Ti metal-2 and 0.76 for Ti alloy-2), indicating substrate-dependent defect density (Table 3). The D and G peaks broadened for all samples, possibly due to defectiveness of the materials and multiwall graphitic structures. The nanoflakes in the Ti metal-3 sample (Figure 5f) comprised multilayer nanosheets, and the nano-rods in the Ti alloy-3 sample (Figure 6f) had thicknesses of 70–140 nm. Additionally, a comparison between the Ti alloy-2 and Ti alloy-3 samples showed a wider D peak (with a shoulder around 1120 cm^−1^), which may have been due to amorphous carbon and a low-frequency shift in the well-pronounced G′ peak for Ti alloy-3, related to multilayer nano-rods (Figure 10b,d).

## 4. Discussion

The surfaces of the samples from both materials demonstrated different morphologies after the grinding and etching steps of sample preparation. The surface of the pure Ti metal possessed a combined micro/nano-roughness and oxide formations (Figure 2b), while the alloy surface had micro/nano-roughness and a small amount of oxide formations along the boundaries between the α- and β-phases (Figure 2d). The different chemical compositions and surface morphologies of the initial substrate create distinct conditions for formation of the nanostructures, resulted in tuning of the surface layer which was growing during the subsequent treatments.

### 4.1. TiO_2_ Nanotubes Coating

After anodization, the morphology of the titanium oxide layer on the surface of pure Ti metal differed from that of the Ti6Al4V alloy. The morphology of the TiO_2_ layer on the Ti metal was mixed, consisting of a “sponge-like” structure, nanotubes, and nano-rods (Figure 4(a–a2) and Figure 5a,b). On the other hand, the morphology of the oxide layer on the Ti alloy was uniform, consisting of mainly nanotubes (Figure 4(b–b2) and Figure 6a,b). Such heterogeneous morphology of the Ti surface was due to the different rates of the oxidation–reduction processes during anodization and the different nanotube formation mechanisms on the surfaces of the pure Ti and Ti alloy [14]. Based on the XRD data, before annealing, the as-prepared coatings were supposed to be amorphous (Figure 7c and Figure 8c) and to consist of nanotubular TiO_2_, which is the main component on the Ti metal surface (Table 2). Additionally, on the Ti6Al4V alloy, mixed-metal (Ti, Al, V) oxides were formed (Table 2), a fact that has been previously confirmed by other researchers as well [42,43]. During the oxidation of the metals, an oxide layer with a composition depending on the matrix alloy was obtained [15]. Decha-umphai D. et al. found different chemical compositions of the TNTs on the surfaces of the α- and β-phases of the alloy [52]. The β-phase was enriched with vanadium due to the accumulation of V at the grain boundaries, while the α-phase was enriched with Al. This is why the surface layer on the α-phase consisted of a mixture of TNTs and aluminum oxide, while that on the β-phase contained TNTs and vanadium oxide. This phenomenon reflects the height of the nanotubes: the β-TiO_2_ nanotubes were shorter compared to the α-TiO_2_ nanotubes. We observed well-shaped TNTs on the α-phase and seeds of TNTs on the β-phase of the Ti6Al4V alloy (Figure 4(b1) and Figure 6a,b).

The annealing process was used to achieve crystallization of the oxide layer and to eliminate the surface fluorine in order to improve the cell responses [43]. It was found that the nanotubular structures on the Ti alloy were stable up to 700 °C [43,44]. In our work, during the annealing, the morphologies of the surface layers of both materials were retained, and at the same time, transformation of the amorphous TiO_2_ to crystalline anatase and rutile occurred, as was proven by Raman spectra (Figure 9). The anatase phase is usually obtained at annealing temperature 450–600 °C, while rutile phase is associated with higher temperature [43,44,52]. The biocompatibility of a mixture anatase/rutile as well as anatase alone is higher compared with the amorphous structure of the oxide layer [43,53]. The growth of anatase crystals at temperature 450 °C occurs mainly along the length and curvatures of the TNTs thus making the morphology stable [54]. The annealing process leads to thicker walls of the oxide nanotubes due to the diffusion of Ti ions along the nanotube’s walls causing subsequent oxidation [52,54]. Thus, the TiO_2_ nanotube’s internal diameter decreases from 120 nm to 85 nm after annealing of the sample Ti metal-1 (Figure 4(a1) and Figure 5b). In contrast to Ti metal-1, which contained anatase and rutile, the other two samples, Ti metal-3 and Ti alloy-3, underwent a complete conversion of anatase to rutile during the CVD process.

### 4.2. CNTs Coating

Our goal was to synthesize CNTs using a catalyst-free CVD process in order to avoid transition metal impurities on the titanium surface. As was previously found, oxides such as SiO_2_ and porous Al_2_O_3_ can facilitate the growth of CNTs [39]. Related to that, our hypothesis was that TiO_2_ could play a similar role and could act as a site for CNT nucleation. On the other side, titanium as a transition metal may act as a catalyst in CNT formation, like other well-known metal catalysts (Fe, Ni, Co) [32,33]. The results of the present study confirmed that the metal-catalyst-free CVD process is capable of producing CNTs on the surfaces of etched Ti metal and Ti6Al4V alloy. The surfaces of both materials were covered with long, curly CNTs when a one-hour CVD process was applied (Figure 5c,d and Figure 6c,d). Most probably, the TiO_2_ oxide nanopeaks from the rough surface of the substrate served as sites for the formation and growth of CNTs.

### 4.3. Bilayer of TiO_2_ Nanotubes/Carbon Nanostructure Coating

We found that the anodized surfaces of both materials were covered with different CNS formed in a one-hour CVD process, namely, CNFs on the Ti-metal-3 and CNRs on the Ti-alloy-3 (Figure 5f and Figure 6f). The distinct D and G peaks in the Raman spectra (Figure 10) are clear evidence for the formation of carbon nanostructures. We could claim that the observed dense layer decorated with nanoflakes on the Ti metal-3 surface (Figure 5e,f) consisted of multilayer carbon nanosheets. A similar surface morphology was reported by Wang et al., who synthesized graphene nanoflakes directly onto ZnO films using a hot filament CVD process, where the graphene structures were vertical to the substrate surfaces [55]. According to Bo et al., the growth mechanism of the vertically-oriented graphene starts with the formation of a buffer layer of amorphous carbon, with irregular cracks serving as nucleation sites [56]. It is well known that graphene layer(s) can be deposited onto the transition metal (Cu, Ni, Pt, Pd, Ir, Ru, Co, Au) surface via the thermal decomposition of a hydrocarbon during the CVD process [57]. Moreover, a graphene monolayer can also be synthesized on insulating surfaces like MgO and SiO_2_ [58,59]. Similarly, in the Ti metal-3 sample, the mixed-structured TiO_2_ layer which formed during anodization may serve as a suitable site for the deposition of the carbon layer in the CVD process. Thus, the high surface roughness and mixed morphology of the anodized Ti metal surface resulted in a buffer carbon layer with high defectiveness, which promoted the growth of vertical CNFs, as shown in Figure 5f.

Furthermore, it was found that binary alloys can suppress carbon precipitation during the CVD process, thus inhibiting graphene monolayer growth [60,61,62]. Our study shows that CNRs were grown directly on the TiO_2_-nanotube walls in the Ti alloy-3 sample (Figure 6e,f). As opposed to the Ti metal-3, a dense carbon layer could not form on the alloy’s surface during the CVD process, because the growth of the graphene monolayer was restricted. On the other hand, the anodized alloy surface was covered with homogeneous nanotubes of mixed oxides, which may have catalyzed the thermal decomposition reaction of acetylene, and various defects in the nanotube’s walls may have served as sites for the nucleation and growth of thin carbon nano-rods (Figure 6f). Therefore, the bilayer coatings on both materials had different mechanisms of carbon nanostructure growth and, thus, different morphologies, showing carbon nano-flakes on the Ti metal-3 and carbon nano-rods on the Ti alloy-3 surfaces.

## 5. Conclusions

The present paper deals with the investigation of the morphology and structure of new coatings based on TiO_2_ nanotubes and carbon nanostructures on pure Ti Grade 2 and Ti6Al4V alloy. Three types of coatings were formed on the surfaces of the titanium materials by using different methods. The TiO_2_ nanotubes were obtained via anodization, the carbon nanotubes were achieved using a metal-catalyst-free CVD process, and the bilayer coating consisting of a TNTs layer covered by carbon nanostructures was prepared through the successive processes of anodization and CVD. 

It was found that the chemical compositions and different surface morphologies of the two materials defined the formations of the surface layers during the successive treatments. After anodization, the morphology of the TiO_2_ layer on Ti metal consisted of “sponge-like” structures, nanotubes, and nano-rods, while the morphology of the oxide layer on the Ti alloy was more homogeneous, containing mainly nanotubes of TiO_2_ accompanied by vanadium and aluminum oxides. The subsequent annealing preserved the morphology of the surface layers of both materials, and simultaneously induced a change in the amorphous TiO_2_ to a crystalline anatase and rutile phase.

Our findings reveal that carbon nanotubes can be successfully synthesized on the rough surfaces of etched pure Ti Grade 2 and Ti6Al4V alloy using a metal-catalyst-free CVD process. Using the latter method, we also prepared carbon nanostructures grown over the TNTs obtained by anodization.

The bilayer coatings on the pure Ti Grade 2 and Ti6Al4V alloy had different morphologies due to the composition of the substrate and the morphology of the TiO_2_ sublayer. In the case of pure Ti Grade 2, the first layer of the TiO_2_ nanotubular and nano-rod structures was covered with a dense carbon layer decorated with vertically oriented carbon nano-flakes. The coating of the Ti6Al4V alloy comprised TiO_2_ nanotubes capped with numerous carbon nano-rods. 

The novelty of this research lies in the formation of two new types of coatings on titanium surfaces: carbon nanotubes synthesized using a metal-catalyst-free CVD process and a hybrid TiO_2_ nanotube/carbon nanostructure bilayer coating. In our upcoming project, we will focus on conducting biological tests on the synthesized materials to gain insight into their potential biomedical applications. 

## Figures and Tables

**Table 1:** Treatment conditions for titanium samples.

Samples			Anodization	CVD Process	Annealing
Material	Treatment	Notation			
	Group 1	Ti metal-1′	25 V, 7 h ^1^		
Pure Ti	Group 1	Ti metal-1	25 V, 7 h		550 °C, 2 h, argon
Grade 2	Group 2	Ti metal-2		650 °C, 1 h	550 °C, 2 h, argon
	Group 3	Ti metal-3	25 V, 7 h	650 °C, 1 h	550 °C, 2 h, argon
	Group 1	Ti alloy-1′	30 V, 7 h ^1^		
Ti6Al4V	Group 1	Ti alloy-1	30 V, 7 h		550 °C, 2 h, argon
alloy	Group 2	Ti alloy-2		650 °C, 1 h	550 °C, 2 h, argon
	Group 3	Ti alloy-3	30 V, 7 h	650 °C, 1 h	550 °C, 2 h, argon

^1^ The samples Ti metal-1′ and Ti alloy-1′ were only anodized, while the samples Ti metal-1 and Ti alloy-1 were anodized and annealed.

**Table 2:** EDX data for the anodized pure Ti metal and Ti alloy before annealing (Ti metal-1′ and Ti alloy-1′) and the pure Ti metal and Ti alloy treated with anodization and CVD process (Ti metal-3 and Ti alloy-3).

Ti Metal-1′	Ti Alloy-1′	Ti Metal-3			Ti Alloy-3
		1	2	3	
Ti 74.67 ^1^	Ti 53.85	Ti 39.27	39.42	45.53	Ti 38.65
O 25.35	O 39.80	O 46.41	42.71	38.65	O 36.97
	Al 5.78	C 14.32	17.86	15.81	Al 4.61
	V 0.57				V 0.00
					C 19.76

^1^ Element content in atomic percentages (at.%).

**Table 3:** Raman data for the coatings on the surface of pure Ti Grade 2 and Ti6Al4V alloy after different treatments.

Sample	Raman Shifts, cm^−1^									
	TiO_2_						Carbon Nanostructures			
	A	A	R	A	R	A	D	G	G′	I_D_/I_G_
Ti metal-1	144	394	442	515	611	635				
Ti metal-2							1308	1596	2692	0.84
Ti metal-3			448		611		1332	1602	2604	0.82
Ti alloy-2							1344	1601	2650	0.76
Ti alloy-3			448		611		1340	1605	2207	0.78

Note: Ti alloy-1 is not shown due to the low quality of the spectrum. A and R stand for anatase and rutile.

## Data Availability

Data are contained within the article.
